# Technical and clinical considerations for electroencephalography-based biomarkers for major depressive disorder

**DOI:** 10.1038/s44184-023-00038-7

**Published:** 2023-10-25

**Authors:** Leif Simmatis, Emma E. Russo, Joseph Geraci, Irene E. Harmsen, Nardin Samuel

**Affiliations:** 1 Faculty of Medicine, University of Toronto, Toronto, ON Canada; 2 Cove Neurosciences Inc., Toronto, ON Canada; 3 Department of Pathology and Molecular Medicine, Queen’s University, Kingston, ON Canada

**Keywords:** Neuroscience, Cognitive neuroscience, Neural circuits

## Abstract

Major depressive disorder (MDD) is a prevalent and debilitating psychiatric disease that leads to substantial loss of quality of life. There has been little progress in developing new MDD therapeutics due to a poor understanding of disease heterogeneity and individuals’ responses to treatments. Electroencephalography (EEG) is poised to improve this, owing to the ease of large-scale data collection and the advancement of computational methods to address artifacts. This review summarizes the viability of EEG for developing brain-based biomarkers in MDD. We examine the properties of well-established EEG preprocessing pipelines and consider factors leading to the discovery of sensitive and reliable biomarkers.

## Introduction

The lifetime incidence of Major Depressive Disorder (MDD) is estimated to be 12% in men and up to 20% in women^1^. In addition to the immense personal and social impact of this disease, untreated or refractory MDD represents a major societal challenge. In 2018, the economic burden of adults with MDD was $326.2 billion USD^2^. The standard treatments for depression include combinations of psychotherapy and pharmacotherapy. Newer therapies, including psilocybin and ketamine or intranasal esketamine, are among several compounds being evaluated for their ability to treat MDD, particularly in treatment-resistant depression^3–5^. While novel treatment strategies are emerging for MDD, objective biomarkers of disease progression and treatment response are lacking. Inadequate biomarkers are in part due to MDD diagnostic criteria, which are primarily behavioral and based on patient-reported symptomatology (DSM^6^, or Beck et al., 1996^7^). To develop novel therapies and optimize current treatments, neurophysiologic biomarkers for MDD are urgently needed.

**Fig. 1:**
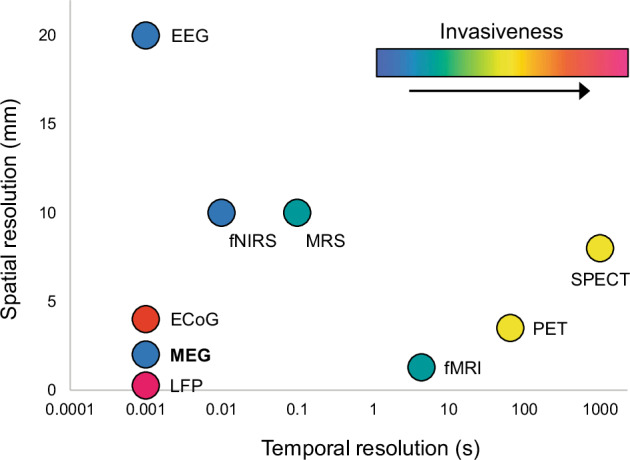
EEG is a noninvasive tool to study the human brain with high temporal resolution but lower spatial resolution. EEG, electroencephalography; ECoG, electrocorticography; LFP, local field potential; fNIRS, functional near-infrared spectroscopy; MRS, magnetic resonance spectroscopy; fMRI, functional magnetic resonance imaging; PET, positron emission tomography; SPECT, single-photon emission computed tomography. Figure adapted with permission from^97^ (Fig. 1).

Various approaches exist to measure local and global neurophysiologic changes in neurologic or psychiatric diseases. These include positron emission tomography (PET), functional magnetic resonance imaging (fMRI), functional near-infrared spectroscopy (fNIRS), magnetoencephalography (MEG), and electroencephalography (EEG). These modalities all markedly differ with regard to the temporal and spatial resolution of the derived signals (see Fig. 1). Among these approaches, EEG may be the most promising tool for biomarker development owing to its accessibility and temporal signal resolution, permitting analyses of neuronal oscillations on the order of milliseconds. Other advantages of EEG include its noninvasive administration, such that patients are not exposed to radiation, high magnetic fields, or noise. Furthermore, EEG devices are portable, which allows for easy setup. Compared to most other imaging or electrophysiological modalities, EEG devices are relatively affordable, and commercial EEG apparatuses are now widely available (Emotiv, San Francisco, USA). Low-cost data collection using EEG is ideal for studies requiring many participants^8^. EEG is ideal for measuring clinically relevant changes in brain activity in neurologic and psychiatric diseases, particularly MDD, in which diagnosis and treatment response are determined clinically.

EEG studies can involve event-related (i.e., task-based) and resting-state recordings. Event-related potentials (ERP) are well-established markers of brain responses to external stimuli such as sensory, cognitive, or motor events^9^. ERPs reflect the cumulative activity of postsynaptic potentials produced by the synchronous firing of cortical pyramidal neurons^10^. P300 is the most widely studied ERP, referring to increased activity about 300 milliseconds after a stimulus, and reflects attention and working memory processes. Another EEG-based ERP includes the loudness dependence of auditory evoked potentials (LDAEP), which measures amplitude changes of auditory evoked potentials in the primary auditory cortex^11^. It is a surrogate of serotonergic activity (e.g., mood, appetite, sleep). Additionally, mismatch negativity (MMN) is an ERP component generated when a sequence of uniform stimuli is interrupted by the infrequent presentation of deviant stimuli. MMN is an important marker in sensory memory and attention^12^. In contrast, resting-state EEG data is collected from patients not engaged in a task and is thought to reflect inherent spontaneous neural activity^13^. Resting-state EEG is used to examine functional connectivity networks, which provide an index of the relationships between brain regions. Traditionally, EEG functional connectivity is measured using estimates of correlation or coherence between neural signals recorded from multiple electrodes^14,15^. EEG alpha asymmetry is also widely investigated and is characterized by an asymmetrical alpha-band (8–12 Hz) in the left and right hemispheres and is known to reflect cortical activity.

This review critically appraises the role of EEG in MDD. Given the ongoing evolution in our understanding of the pathophysiology of MDD from a purely chemical imbalance to one incorporating multifactorial processes, including abnormal brain circuitry, techniques that quantify brain activity become increasingly valuable^16^. As such, EEG represents a promising modality for discovering and clinically validating biomarkers for MDD.

## EEG signatures of MDD

A diagnostic biomarker detects or confirms the presence of a disease, identifies an individual with a disease subtype, and can be used to evaluate pharmacological effects (FDA Biomarkers, EndpointS, and other Tools (BEST) Resource^17^,). To grant clinical utility, a biomarker must be specific (i.e., accurately detect patients who do not have the disease) and sensitive (i.e., identify the presence of disease in patients who do have the disease)^18^. Commonly used clinical biomarkers include glomerular filtration rate (GFR) used to detect kidney disease^19^, hemoglobin A1c to diagnose diabetes^20^, and blood pressure to detect essential hypertension^21^. Establishing the clinical use of a biomarker requires fulfillment of several criteria, including the demonstration that the biomarker is significantly different in diseased patients compared to control, the assessment of the diagnostic properties of the biomarker, and the comparison of the diagnostic properties of the biomarker to existing tests^18^. EEG has already fulfilled the initial requirements necessary to be regarded as a potential biomarker for MDD.

Many studies have demonstrated the utility of EEG in detecting changes in neural activity in patients with MDD. For example, alpha band functional connectivity in the default mode network (DMN) can predict depression severity^22^ and is more prominent in MDD patients than healthy controls. Subjects with MDD also had higher clustering coefficients and local efficiency in both the alpha and beta bands^23^. Furthermore, multi-dataset cross-validation demonstrated that patients with MDD had decreased amplitude envelope correlation (AEC), a measure of signal coupling (functional connectivity), within the beta band compared to healthy controls^24^. These findings support the use of resting-state connectivity between the subgenual anterior cingulate cortex (sgACC) and the dorsolateral prefrontal cortex (DLPFC) to measure disease severity. In a study measuring phase lag, another functional connectivity metric, authors observed an increase in coupling between two regions of the DMN (right superior frontal gyrus and right parahippocampal gyrus)^25^. DMN connectivity to the central executive network (CEN) was also greater in patients with MDD compared to healthy controls or patients in remission of MDD. Moreover, patients with MDD had increased delta-band phase lag at baseline compared to healthy controls, which decreased following a musical stimulus^26^. Patients with MDD also have reduced resting-state gamma current density in the anterior cingulate cortex^27^ and increased resting-state complexity of gamma signaling in the frontal and parietal cortex^28^. See Table 1 for a summary of MDD-related EEG findings^29–31^.

**Table 1:** MDD-related EEG findings in the literature.

Findings	Brain regions/Networks implicated	Source	EEG Paradigm
Alpha band functional connectivity increased in MDD	Default Mode Network (caudal middle frontal gyrus, insula, parahippocampal, posterior cingulate, rostral anterior cingulate)	^22^	Dataset 1: resting state for 5 min Dataset 2: resting state for 5 min, EC Dataset 3: resting state for 16 min, EO and fixated on a low contrast cross on a gray background
Alpha and beta band clustering coefficient and local efficiency	Left lingual gyrus and left precuneus	^23^	Resting-state for 4 min, EC
Beta AEC functional connectivity increased in MDD	sgACC and DLPFC	^24^	Resting-state 12-min segment, EO for 6 min, and EC for 6 min; only EC segments analyzed
Phase lag increased in MDD	Within the Default Mode Network (right superior frontal gyrus and right parahippocampal gyrus); Between Default Mode Network (left superior frontal gyrus) and Central Executive Network (right middle temporal gyrus)	^25^	Resting-state, eight 1-min segments, 4 segments EO, and 4 segments EC; only EC segments analyzed
Delta phase lag and theta phase lag	Delta phase lag decreased in MDD in the frontal pole, frontal lobe, and parietal lobe after musical stimulus; theta phase lag increased in MDD in the central region after musical stimulus	^26^	3 segments for a total of 6 min Part 1: resting state for 1 min Part 2: Music audio played for 4 min Part 3: resting state for 1 min
Resting gamma current density in MDD	Increased in the posterior cingulate cortex (Brodmann Areas 23 and 31) and decreased in Brodmann Area 25	^27^	Resting-state for eight 10-min trials, 4 EC and 4 EO
Resting gamma complexity increased in MDD	Frontal and parietal cortex	^28^	
Gamma during arithmetic counting and spatial imagination tasks increased in MDD	Frontal, anterior frontal, and temporal areas of the cortex symmetrically, and in the central and parietal temporal areas of the left hemisphere	^98^	Resting-state and cognitive task consisting of arithmetic counting and spatial imagination while EC for 100 seconds
Alpha power decreased in MDD	Areas corresponding to electrodes Fp1, Fp2, F3, F4, F7, F8, C3, C4, P4, O2, T3, T4, and T6 in the international 10–20 electrodes placement system	^32^	Resting-state for 2 min EC
Alpha power increased in MDD	Frontal and parietal regions	^33^	Resting-state for 3 min EC and 3 min EO
Alpha power increased in MDD	Frontal and parietotemporal regions	^34^	Resting-state for 2 min, EC

*AEC* amplitude envelope correlation, *DLPFC* dorsolateral prefrontal cortex, *EC* eyes closed, *EO* eyes open, *MDD* major depressive disorder, *sgACC* subgenual anterior cingulate cortex.

Despite discovering many potential indicators of MDD, there are some conflicting reports. For example, when analyzing the alpha band in MDD^32^, demonstrated that patients with MDD have decreased alpha power compared to healthy controls. In contrast, it has also been shown that alpha power is increased in MDD^33,34^. For more details, see Table 1.

This is by no means a comprehensive list of the literature regarding differences in healthy individuals compared to those with MDD in the context of EEG. For reviews dedicated solely to discussing these differences, readers are directed to manuscripts such as De Aguiar and Rosa, 2019,^29–31^. It should also be noted that further investigation is needed, given the limitations in validating findings across other studies. Inconsistent results arise from differing methodologies, including the frequency range used for alpha or gamma bands^29,31,35,36^, eyes open versus closed^37,38^, diagnostic criteria (e.g., DSM^6^, or Beck Depression Inventory, Beck et al., 1996^7^), and remission criteria^37–39^.

## EEG in MDD subtyping and prediction of treatment response

MDD is a heterogeneous disorder with multifactorial etiologies^40^. In addition to the lack of objective measures of disease progression or treatment response, there is a shortage of objective approaches to stratify MDD. An understanding of MDD subtypes is essential to tailor treatment approaches accordingly. Recent studies have shown that EEG may aid in distinguishing subtypes of MDD. Zhou et al.^41^, used EEG to demonstrate three subtypes of MDD based on alpha and beta left-right asymmetry in the prefrontal lobe, wherein main clinical symptoms differed between groups. In another study, EEG resolved two MDD subtypes with distinct functional connectivity patterns without clinical differences^42^. Of the two subtypes, one included significantly more responders to sertraline than the other. These findings demonstrate that objective measures of disease subtype may be used to predict and optimize treatment response.

Although several classes of pharmacological treatments for MDD exist, only 60–70% of patients respond adequately to two trials of different classes of antidepressants, after which patients are considered to have treatment-resistant depression^43^. Several studies suggest that EEG can predict which patients with MDD benefit from conventional medications. A meta-analysis by^44^ on treatment response prediction using EEG reported accuracies of 85.7% for estimating response to repetitive transcranial magnetic stimulation (rTMS) and 81.4% for estimating response to antidepressants. Another study demonstrated a negative correlation between baseline resting-state EEG connectivity of the right-lateralized frontotemporal network and response to SSRI treatment after 2 months^45^. Moreover, EEG may also be used to predict placebo response. For example^46^, demonstrated that greater alpha-band power envelope connectivity (PEC) within parietal, temporal, and visual regions predicted better treatment outcomes with placebo but not sertraline.

In addition to the general prediction of treatment response, EEG has the potential to predict sex-specific treatment response. For example, an association between better treatment response and higher right frontal alpha power was observed after two months in females with MDD but not males^38^. Arns et al.^39^, showed similar findings, such that SSRI response and MDD remission were associated with greater right frontal alpha in females only for both eyes open and closed conditions. Predicting treatment responses with EEG may allow patients and clinicians to make more informed and personalized decisions regarding the best treatment plan.

## Using EEG to distinguish MDD from other psychiatric conditions

Individuals with MDD often suffer from comorbid psychiatric disorders, including generalized anxiety disorder, substance use, and post-traumatic stress disorder. Determining a patient’s correct diagnosis is essential to guide clinical decision-making. In addition to differentiating patients with and without MDD, EEG has been used to discriminate between MDD and other psychiatric disorders. In a study examining frontal alpha and theta activity in response to emotional face stimuli in individuals with bipolar disorder (BD), MDD, or healthy controls, changes in theta activity distinguished BD from MDD^47^. Tas et al.^48^, showed that patients with BD, compared to those with MDD, had greater discordant activity in the right parietal cortex, greater central-temporal theta coherence, and greater parietal-temporal alpha and theta coherence. Compared to individuals with somatic symptom disorder (SSD), patients with MDD had greater theta coherence in the inferior frontal gyrus, dorsolateral prefrontal cortex, angular gyrus, and supramarginal gyrus^49^. Lastly^50^, distinguished between MDD patients with and without comorbidities. Individuals with MDD and internet gaming disorder had less alpha coherence in bilateral frontal regions than individuals with MDD alone.

Combining factors, EEG may predict treatment response to MDD and differentiate MDD from other disorders. Examining the response to rTMS^51^, compared four patient groups: MDD-responders, BD-responders, MDD-non-responders, and BD-non-responders. MDD-responders had greater delta and gamma activity before and after stimulation compared to BD-responders. In addition, alpha activity in the left frontal and right centroparietal areas was lower in MDD-non-responders compared to BD-non-responders.

Despite these promising results, the limitations of EEG must be considered. EEG spatial resolution is well known to be inferior to MRI^52^ due to limited spatial sampling or contamination of the reference electrode^53^. The skull also distorts the underlying electrical activity of the brain over large areas of the scalp^54^. This phenomenon, known as volume conduction, leads to an electrical field detected at multiple electrodes that may not be near the original dipole^55^. Olbrich & Arns^56^, also describe several challenges regarding inadequate standardization of EEG experiments, which may hinder progress toward clinically useful and valid biomarkers for MDD. Some examples include discrepancies in the length of recordings and data epochs, the definition of regions of interest, recording environment (e.g., light, noise, temperature, time of day), and variability in preprocessing pipelines (e.g., differences in artifact correction or removal).

## Technical considerations for EEG analysis pipelines

**Fig. 2:**
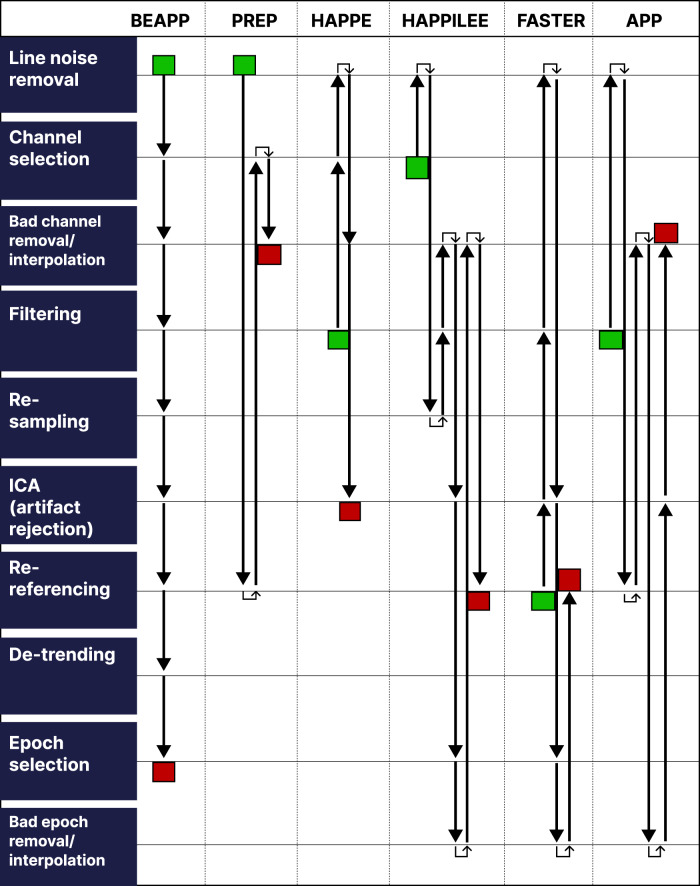
Depiction of the processing steps in some commonly used EEG analysis pipelines. Green boxes indicate the start of the pipeline, and red boxes indicate the end of the pipeline. Boston EEG Automated Processing Pipeline (BEAPP) is used as a reference pipeline because it is the most comprehensive in terms of processing steps used. APP, Automatic Pre-processing Pipeline; BEAPP, Boston EEG Automated Processing Pipeline; FASTER, Fully Automated Statistical Thresholding for EEG artifact Rejection; HAPPE, Harvard Automated Processing Pipeline for Electroencephalography; HAPPILEE, HAPPE in Low Electrode Electroencephalography; ICA, Independent Component Analysis.

Given the strong rationale for using EEG in the clinical setting, attention should be paid to developing and validating EEG data processing and analysis pipelines. EEG processing pipelines are numerous due to the substantial diversity of research objectives and the potentially large number of processing steps (Fig. 2). Developing EEG analysis pipelines for depression will require careful attention to design choices to ensure that robust and effective biomarker pipelines can be created. This section will provide an overview of some of the most common processing steps used in EEG pipelines and contrast technical considerations of variants of each processing stage.

A series of common processing steps during the development of the Boston EEG Automated Processing Pipeline (BEAPP) has been previously described by^57^ and is explored in more detail here. These steps include line noise removal and filtering, resampling, independent components analysis (ICA) for artifact rejection, channel selection/removal, re-referencing, epoch selection, and final output generation. There are numerous factors affecting analytical choices at each of these steps. Although innovation is at various stages throughout this process, the field has converged towards a general set of criteria over the past decade that appears to balance robustness, efficiency, and ability to integrate with existing software interfaces.

Early-stage preprocessing typically consists of filtering and possible line noise removal (depending on frequency ranges of interest). These are important steps for preventing overt environmental contamination of true brain-derived signals by electrical line noise at 50 or 60 Hz and muscle artifact. The first challenge to address is line noise, which^58^ demonstrated could dramatically influence connectivity estimates. Several popular methods exist to reduce line noise, each with specific strengths and limitations. The simplest filter option is a notch or bandstop filter that selectively removes specific frequency bands from the overall signal. Luck^59,60^ cautioned against applying notch filters and suggested that low-frequency high-pass filtering may induce undesirable signal distortions in frequency bands of interest. Leske and Dalal^61^ suggest that spectrum interpolation may be more robust for removing line noise with variable amplitude. Alternatively^62^, demonstrated in their PREP pipeline that other methods, such as CleanLine, were robust if detrending was applied. The same group also cautioned against using low-frequency (1 Hz) high-pass filters before removing line noise due to concerns over the impact on downstream connectivity analyses^62^.

An additional early preprocessing concern is electromyography (EMG) noise, which is more difficult to solve than line noise, given that EMG and EEG may overlap. EMG noise typically occurs at higher frequencies than EEG noise; therefore, a 30 Hz low-pass filter could resolve the issue^59^. However, Goncharova et al.^63^ noted that there is variability in the frequency ranges of EMG noise that depends on the specific scalp region under consideration. For example, frontal EMG noise may be as low as 20 Hz, whereas temporal EMG noise occurs at >40 Hz. Fortunately, Zhou and Gotman^64^ demonstrated that applying wavelet thresholding and ICA might be sufficient to remove EMG contamination. ICA can also address other signal quality challenges by simultaneously removing EMG.

Next, “bad” channels and epochs resulting from various technique-related reasons (e.g., cap shift, sweat, poor cap fit) are identified and then interpolated or removed. Typically, channel issues relate to global problems with an electrode, whereas epoch issues arise from transient activity affecting multiple channels, such as head movement. There have been several methods developed to handle each of these cases. The FASTER method developed by^65^ uses a correlation-based criterion (correlation of a channel with its neighbors) and a dispersion criterion (the variance of a given channel) to mark bad channels. Bigdely-Shamlo et al.^62^, used a similar set of statistical criteria based on robust correlation, noise, and a novel method that detects groups of bad channels using the RAndom SAmple Consensus (RANSAC) subset selection approach. In FASTER and PREP, whole channels were spherically interpolated using EEGLAB (Matlab). Newer pipelines, such as the Automatic Pre-processing Pipeline (APP)^66^ and Automagic^67^, built upon these classic methods. They used Artifact Subspace Reconstruction (ASR), Multiple Artifact Rejection Algorithm (MARA), and robust principal component analysis (PCA) for interpolation. Other improvements were proposed by Kumaravel et al.^68^, who used a local outlier factor (LOF)-based approach for bad channel detection. LOF outperformed FASTER and standard statistical methods such as Euclidean distance. Dong et al.^69^ also proposed a high-performance interpolation method called RESIT (Reference Electrode Standardization Interpolation Technique) that yields lower errors and higher correlations with simulated data than popular nearest-neighbor or spherical spline interpolation methods. Although promising, new channel identification methods require further testing and validation.

Another essential step in EEG data processing is artifact rejection to remove eyeblinks or scalp muscle contamination. Although many artifact rejection methods exist^70^, ICA is among the most popular. Briefly, ICA decomposes a signal into individual components (ICs), identifies and removes noisy ICs, and then reconstructs the data with the remaining ICs. ICA can outperform electrooculogram (EOG) regression-based approaches when combined with a 1–2 Hz high-pass filter^71^. The simplicity and effectiveness of ICA have led to its inclusion in automated artifact removal systems such as MARA^72^. Grin-Yatsenko et al.^73^ highlight the influence of ICA on EEG when comparing MDD patients to healthy controls.

Lastly, re-referencing is fundamental in pipeline development and can affect downstream connectivity estimates if not optimized. Common re-referencing methods include average reference (AR), robust average reference (RAR), and reference electrode standardization technique (REST), as well as linked mastoids (LM), which use fewer sensors. Recent work has suggested that REST^74^ or RAR^75^ performs the best among referencing methods. Yang et al.^76^ also suggested that REST is superior when studying event-related potentials. Furthermore, both^77^ and Hu et al.^78^, suggested that REST is preferred over AR in cases where electrode density is not very high. However, REST requires a realistic head model to function, estimated using the finite element method^77^. Mumaz and Malik^79^, suggest that REST may be the most appropriate reference choice for MDD EEG research compared to the link-ear (LE) reference and AR. Many technical considerations for EEG data processing and analysis must be made to optimize data outputs and the development of viable and clinically relevant biomarkers for MDD.

## EEG analysis pipelines and implications for MDD

An important factor in selecting specific processing steps for EEG pipelines for depression is the impact on downstream metrics. Although assessing features based on the “true” EEG signal is difficult, we can evaluate the impact of different processing steps on known datasets and quantify how processing choices affect the psychometric properties underpinning biomarker development and validation. Robbins et al.^80^ demonstrated that different EEG pipelines alter channel-level power estimates. Similarly, other works have explored the effects of various processing strategies on connectivity features.

There has been considerable research on the effects of different head models and inverse solutions on connectivity estimates. For example, Cho et al.^81^ used simulations to study the contribution of head models and source estimation on multiple connectivity measures. They found that modeling the CSF compartment led to significantly fewer errors in source reconstruction and connectivity estimates. Furthermore, source localization was least affected across combinations of datasets and pipelines. In contrast, effective connectivity (generalized partially directed coherence) was most affected, and functional connectivity (imaginary part of coherence) was less so. These findings are particularly relevant to EEG pipelines for depression, given the volume of previous work focused on alpha activity in depression-related functional networks. Anzolin et al.^82^, also identified that the choice of inverse solution (eLORETA vs. LCMV beamformer) impacted Granger causality, albeit for broadband connectivity. More recently^83^, analyzed resting state networks in high-density EEG (256 channels) using different head models to understand the potential impact of random and systematic errors due to sensor placement. They reported differences between MRI-guided models and template head models rather than between different templates. More specifically, a 3-tissue template-based model performed comparably to a 12-tissue template model, although both performed worse than the MRI-guided model. Thus, EEG connectivity pipelines for MDD should strive to use MRI-guided models; however, head model and alpha band connectivity metrics are robust enough if patient MRIs are unavailable.

## Technical considerations for clinical translation of EEG analysis for MDD

EEG processing can influence the psychometric properties (e.g., test-retest reliability) of connectivity measures, which are, in turn, used for the analytical and clinical validation of biomarkers in MDD. As such, processing steps influencing the reliability and validity of EEG measures have been widely studied. While earlier works established that EEG power analysis can be highly reliable^84^, more recent studies focused on connectivity measures. For example^85^, used simultaneous high-density EEG and MEG to understand the test-retest reliability of source-space network connectivity in 19 healthy individuals. Using the imaginary part of coherence (iCOH) and weighted phase lag index (wPLI) for functional connectivity, they found that reliability was at least fair (intraclass correlation type (1,1) between 0.4–0.59), with the alpha band generating the highest psychometric values. In another study^86^, analyzed the reliability of high-density (256 channel) resting-state EEG in 21 healthy participants using different recording conditions (eyes open vs. closed, 32 vs. 256 channel densities), connectivity estimators (PEC vs. iCOH), and source reconstruction methods (beamformer vs. MNE). Ultimately, they found that alpha band reliability was the highest among all frequency bands. These results are promising for EEG-based connectivity metrics for MDD where alpha activity is of particular interest, mainly because of the conflicting reports regarding the alpha band and MDD, discussed above^32–34^.

Ongoing advancements in EEG analysis may use increasingly automated and end-to-end processing systems such as machine learning to transform raw data. Convolutional neural networks (CNNs) can automatically and adaptively learn and are one of the most widely used deep learning neural networks (e.g.,^87,88^). Already, CNNs have been used to extract features from input EEG to distinguish healthy controls from patients with MDD. Ay et al.^89^, reported classification accuracies of 99.1% and 97.7% for right- and left-hemisphere EEG signals, respectively. In this study, the raw EEG waveforms were applied to the CNN model, and the feature maps obtained from this step were fed to long short-term memory (LSTM) on which learning was performed. Deep learning is yet another method to consider implementing in EEG analysis pipelines for future MDD biomarker development.

## Discussion and concluding remarks

Depression is a debilitating disease at both the personal and societal levels. It is estimated that 322 million people suffer from MDD worldwide^2^. MDD is diagnosed through behavioral analysis since no clinical biomarkers are readily available in practice. Moreover, understanding of MDD pathophysiology has shifted towards the involvement of altered brain activity and connectivity^16^. Therefore, applying devices that can detect neural activity and lead to discovering new biomarkers is timely^16^ for several reasons. In particular, there has been a rapid rise of new EEG-based technologies such as wireless systems^90,91^ and brain-computer interfaces^92^. Furthermore^93^, emphasize the necessity for biomarkers that allow for evidence-based choices pertaining to treatment options for MDD (i.e., personalized medicine). Additionally, it was estimated that there was an increase in cases of MDD by 27.6% worldwide from 2020–2021^94^, thus demonstrating the urgency of this matter.

EEG is the most promising of all neuroimaging and neurophysiological modalities since it is portable, noninvasive, and relatively affordable, allowing large-scale data collection for clinical and/or research purposes. Already, EEG can detect significant differences between patients with MDD and healthy controls^22,24,28^, identify MDD subtypes^41,42^, discriminate between MDD and psychiatric comorbidities (e.g., bipolar disorder)^47,48^, and predict treatment response to antidepressants and rTMS^44,45^.

However, inadequate experimental standardization remains a major limitation of EEG and can hinder the development and clinical translation of MDD biomarkers. To generate robust and effective MDD biomarkers, EEG analysis pipeline development requires careful consideration. Common processing steps include line noise removal to avoid environmental contamination, “bad” channel and epoch identification and subsequent interpolation or removal, artifact rejection, re-referencing, epoch selection, and output generation. New processing features will also arise as EEG analysis incorporates artificial intelligence algorithms to uncover signals from raw data^87^. Ultimately, it is important to recognize that EEG users make choices at each processing step that can affect results. Careful selection can uncover EEG-based biomarkers for MDD to refine diagnoses and personalize treatment strategies to achieve better clinical outcomes.

Other reviews addressing the relationship between EEG and MDD summarize the findings thus far that distinguish between MDD patients and healthy controls at the brain-activity level^31,95,96^. Thus, this highlights the novelty of this review. Herein, we address specific technical considerations researchers and clinicians should evaluate regarding preprocessing steps for EEG analysis of MDD. Since the diagnosis of mental illness strives to be more efficient and accurate, it is becoming increasingly crucial to keep these considerations in mind, particularly because the field of EEG in MDD research lacks a “golden standard” for preprocessing steps. We hope to standardize the field by establishing these frameworks and creating a unified way forward. Further developing a better understanding of state-of-the-art processing techniques also allows for advancements in utilizing EEG to detect biomarkers for MDD. Ultimately, the development of robust biomarkers for MDD will improve therapeutic outcomes for patients worldwide.
